# Effects of color lenses on visual evoked magnetic fields following bright light

**DOI:** 10.1371/journal.pone.0201804

**Published:** 2018-08-02

**Authors:** Masaya Suzuki, Naoya Kumagai, Koji Inui, Ryusuke Kakigi

**Affiliations:** 1 R&D Department, Tokai Optical Co., Ltd., Okazaki, Japan; 2 Department of Integrative Physiology, National Institute for Physiological Sciences, Okazaki, Japan; 3 Institute for Developmental Research, Aichi Human Service Center, Kasugai, Japan; Universidade do Minho, PORTUGAL

## Abstract

Photophobia is a common condition in which bright light causes an unpleasant feeling due to increased sensitivity to light. In addition to discomfort, photophobia may be accompanied by visual dysfunction. The present study was conducted in order to examine whether visual evoked cortical responses contribute to the assessment of visual dysfunction due to bright light. Visual evoked magnetic fields (VEFs) following the presentation of a uniform bright light of 200–3700 cd/m^2^ in the lower visual field were recorded in 10 healthy volunteers and the effects of five color lenses: yellow, blue, gray, green, and colorless, were examined. VEFs were subjected to a multi-dipole analysis that resulted in the separation of several source activities, including the retina, V1, V2, V6, and fusiform gyrus. Source activity in the retina corresponding to the ERG b-wave exhibited a reduced amplitude and elongated peak latency with the yellow lens. Its latency strongly correlated with transmittance at 450 nm. On the other hand, cortical activities in V1 and the fusiform gyrus were stronger with the yellow lens than with the other color and colorless lenses. Only blue-light blocking showed significant effects. The result showing that the yellow lens enhanced V1 and fusiform activities indicated that processing in these areas was improved when subjects used this lens. The combination of delayed retinal activity and increased visual cortex activity may be an objective indicator of the effects of a color lens on visual function.

## Background

Photophobia is a state of increased sensitivity to light in which light feels too bright and uncomfortable. Therefore, photophobia is an internal experience, similar to pain, and its objective assessment is difficult. Photophobia is not a rare symptom; it is associated with various conditions, including migraine, blepharospasm, eye diseases, such as dry eye, and mental diseases, such as panic disorder or depression (for a review, see [1]). Although photophobia is considered to represent abnormal activation in a specific neural circuit, the underlying mechanisms have not yet been elucidated in detail. In addition to the difficulties associated with its objective assessment, the treatment of photophobia using a tinted lens is problematic. Subjects with photophobia wear darkly tinted lenses [2]; however, one principal treatment of photophobia is to decrease the dark adapted state [1]. Therefore, objective assessments and the appropriate use of tinted lens are important. The wavelength of light has been shown to affect photophobia [3,4]. Stringham et al. assessed photophobia using electromyography and showed increased sensitivity to light with decreasing wavelengths [4].

Several neuroimaging studies examined photophobia with a focus on photophobia-related brain activation [5–7]. The findings of these studies provided information on the mechanisms underlying the perception of photophobia, for example, photophobia-induced activation in the so-called pain matrix [6]. In addition to discomfort and pain, photophobia may be accompanied by visual dysfunction, and distinctions must be made between them [8]. In the present study, we focused on the effects of colored lenses on cortical activation in visual areas in order to investigate the mechanisms responsible for visual disturbances caused by bright light. Visual evoked magnetic fields (VEFs) following flash stimuli were recorded in ten healthy subjects and compared among five color lens conditions: yellow, blue, gray, green, and non-color, using a 306-channel whole head MEG system.

## Methods

The experiment was performed on ten (four females and six males) healthy volunteers, aged 25–48 years (35.0 ± 5.8) with normal corrected visual acuity (20/20) and without neurological and ophthalmic disorders. Among the ten subjects tested, one was emmetropic and the remaining nine were myopic. The mean spherical equivalent (S + C/2) was -3.2 ± 1.7 D for the left eye and -3.0 ± 1.5 D for the right. During the experiment, they were corrected to normal vision (20/20) with ophthalmic lenses. The present study was approved in advance by the Ethics Committee of the National Institute for Physiological Sciences, Okazaki, Japan, and written consent was obtained from all subjects.

### Stimulus and recordings

**Fig 1:**
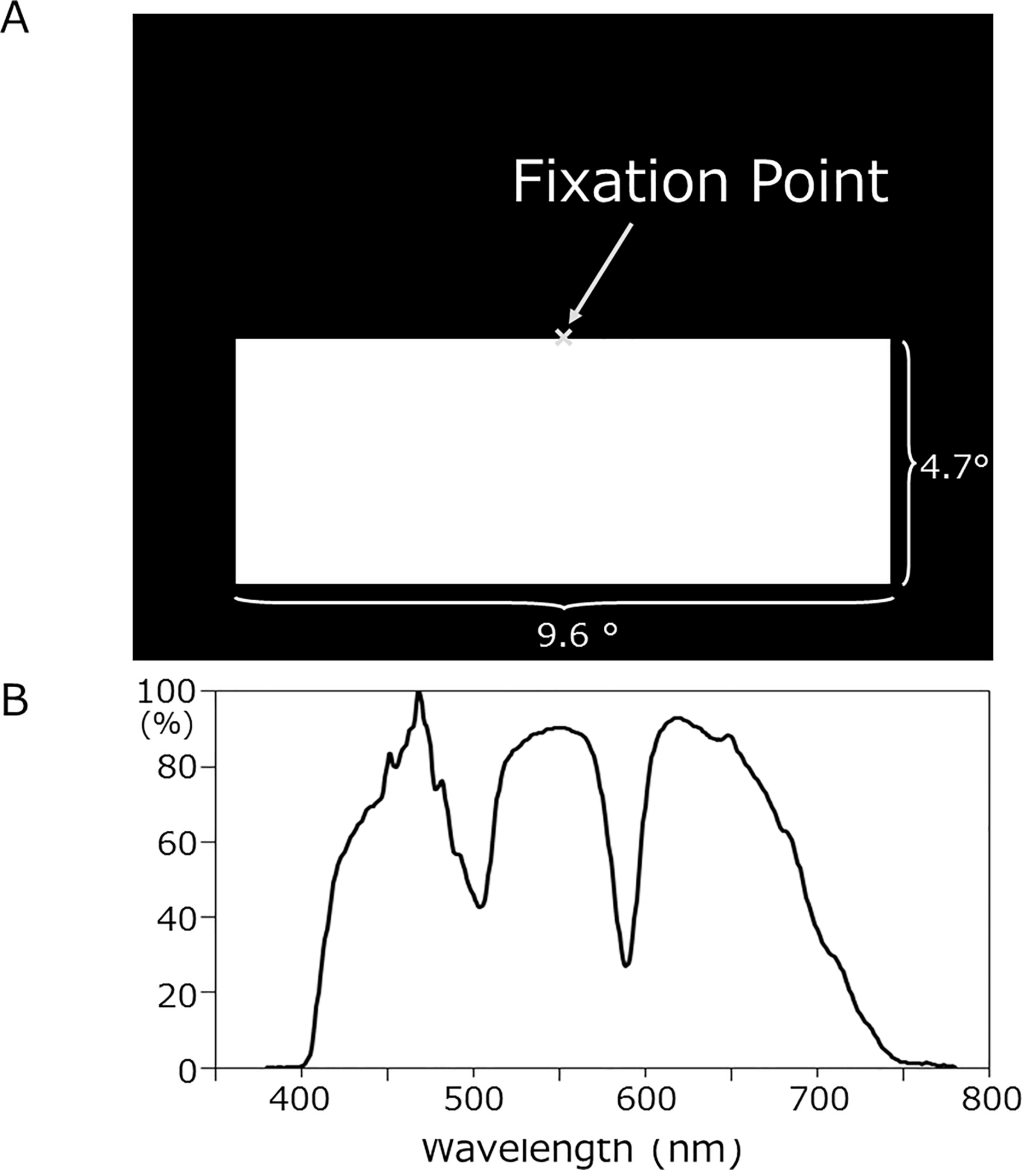
Stimulus. A, Stimulus. B, Relative spectral distribution of the light source.

Since it was not possible to use electric devices in a shielded room because of magnetic noise, the visual stimulus was presented by a digital light processing projector placed outside the shielded room (Mirage 2000, Christie Digital System Inc., Kitcherner, Canada) at an interval of 1575 ms ± 5%. The stimulus was a flash of 300 ms and was applied to the subjects through a small window at a distance of 2 m from the eye. The stimulus was applied to the lower visual field (Fig 1A), which evoked greater VEF responses than the upper visual field [9,10]. Fig 1B shows the power of the flash as a function of the wavelength measured by a spectral radiance meter (CS-2000, Konika Minolta Japan, Tokyo). Clear notches were observed at approximately 500 and 590 nm in the curve, and were attributed to the properties of the projector. VEFs were recorded as described elsewhere [10,11]. Magnetic signals were recorded with a filter of 0.1–200 Hz at a sampling rate of 1004 Hz. The window of analysis was from 100 ms before to 600 ms after the stimulus, and the prestimulus period was used as the DC baseline. Epochs with MEG signals larger than 2.7 pt/cm were rejected from averaging. One hundred epochs without artifacts were collected for each condition.

### Procedures

**Fig 2:**
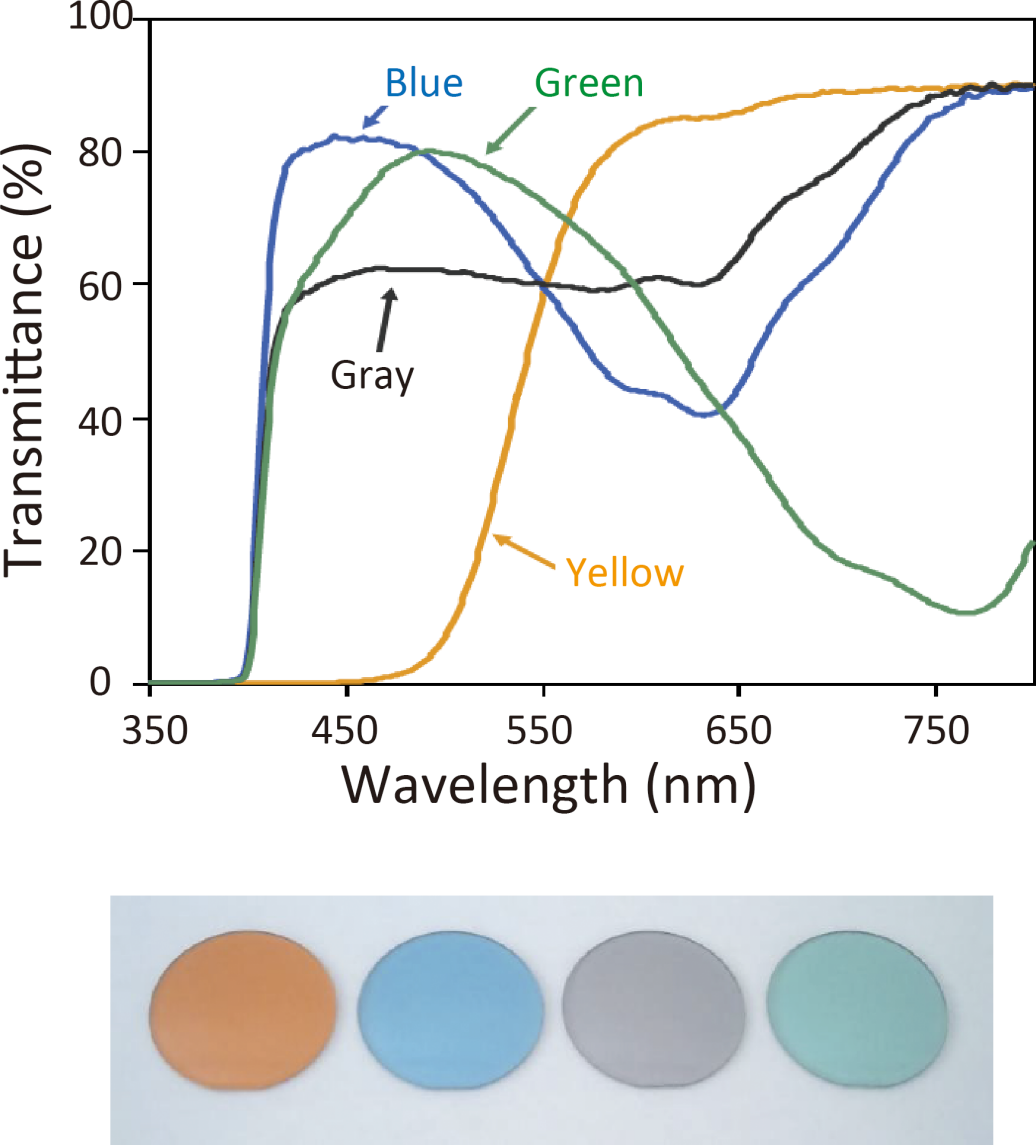
Spectral transmittance curves of color lenses. A colorless lens and four color lenses were used. Luminous transmittances for the color lenses were adjusted to an optical density of 70% of the light source.

In Experiment 1, the effects of the strength of the light on VEFs were examined using stimuli of 3.7 × 10^3^, 1.2 × 10^3^, or 0.2 × 10^3^ cd/m^2^. Stimuli were presented in a random order. In Experiment 2, VEFs following a flash of 3.7 × 10^3^ cd/m^2^ were compared among five lens conditions: yellow (CCP LY, Tokai Optical Co., Ltd.), blue, gray, green, and control (colorless). The luminous transmittance of each color lens was adjusted to 70% of the light source. Fig 2 shows the spectral transmittance curve of each lens. Recordings for each lens condition were performed one by one, and the order was randomized across subjects.

### Analysis

A multi-dipole analysis was performed using the brain electric source analysis (BESA) software package (NeuroScan, Mclean, VA) in order to separate temporally overlapping sources as described elsewhere [11,12]. The location of the estimate dipoles was expressed in Talairach coordinates using BrainVoyager (QX 1.4, Brain Innovation BV, Maastricht, the Netherlands). The latency and amplitude of the source strength waveform obtained were measured for each source activity.

A one-way repeated measures analysis of variance (ANOVA) was used for statistical comparisons of latency and amplitude among three intensity conditions (Experiment 1) or five lens conditions (Experiment 2) followed by paired *t*-tests between the colorless lens and color lenses corrected with Bonferroni adjustments for multiple comparisons. Due to large inter-individual variations in the data obtained, a Wilcoxon signed-rank test was also used in order to confirm the significance of differences. A p value less than 0.05 was considered to be significant. Data are expressed as the mean ± standard deviation.

## Results

### Experiment 1

**Fig 3:**
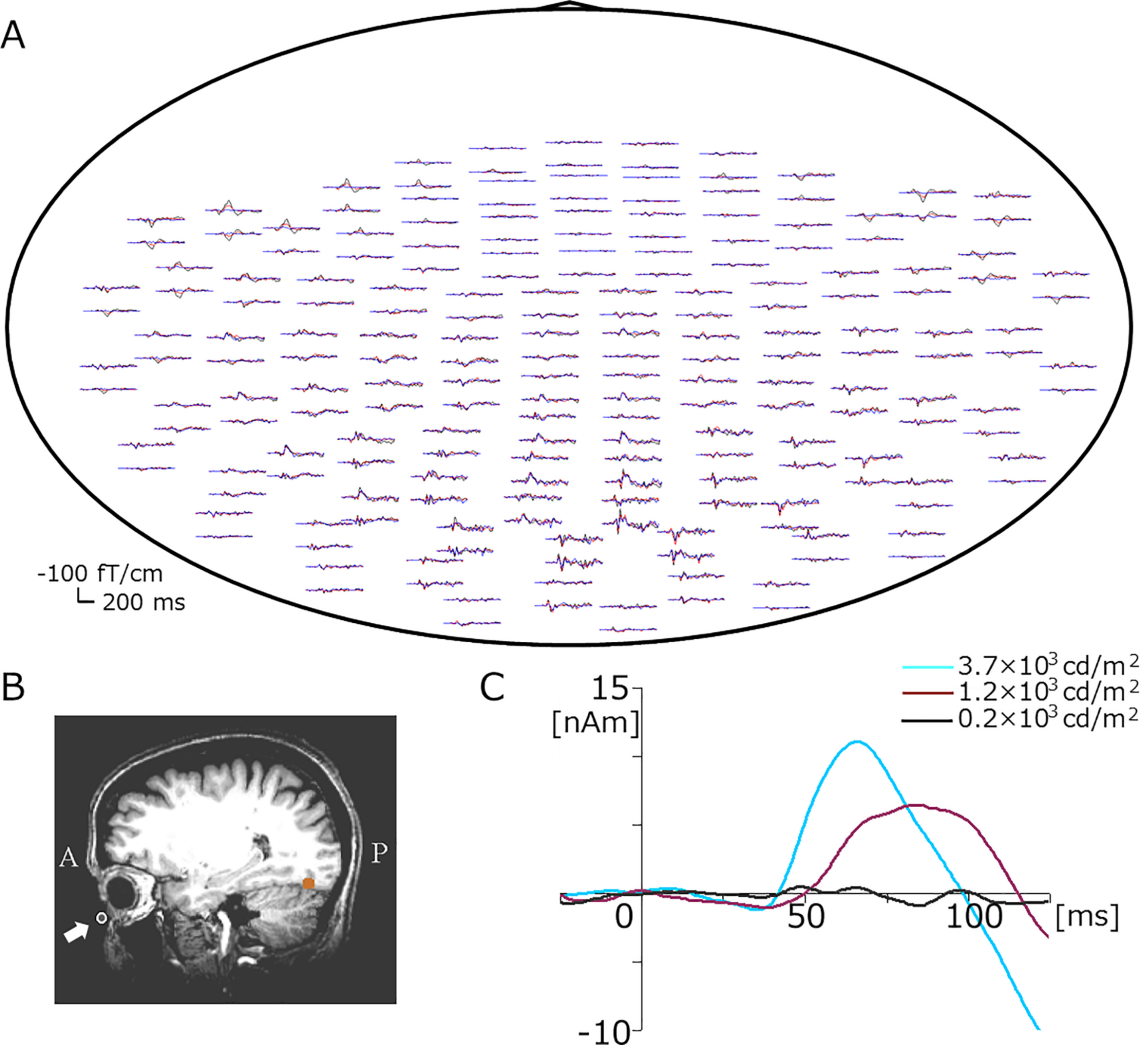
VEFs induced by bright light. A, Top view of MEG waveforms of a representative subject in Experiment 1. B, Location of the estimated dipole in the eye superimposed on a subject’s own MR image. C, Grand-averaged source strength waveforms of retinal activity.

Fig 3 shows an example of VEFs in a subject. Early activities were recorded in the frontal region in addition to the main MEG responses in the occipital area. The results of multi-dipole analyses revealed that early activity to originated from the eye. Based on the location of activation and temporal dynamics with a peak at approximately 60 ms, this activity was considered to correspond to the b-wave of electroretinograms (ERGs). The dipole for this activity was estimated to be located in the cornea in most subjects (Fig 3B). Since the b-wave reflects the positivity of the cornea relative to the posterior pole, the intracellular current in the anterior direction in the present study matched the electric field of the b-wave. As shown in Fig 3C, retinal source activity increased its amplitude with stronger light intensities as expected.

**Fig 4:**
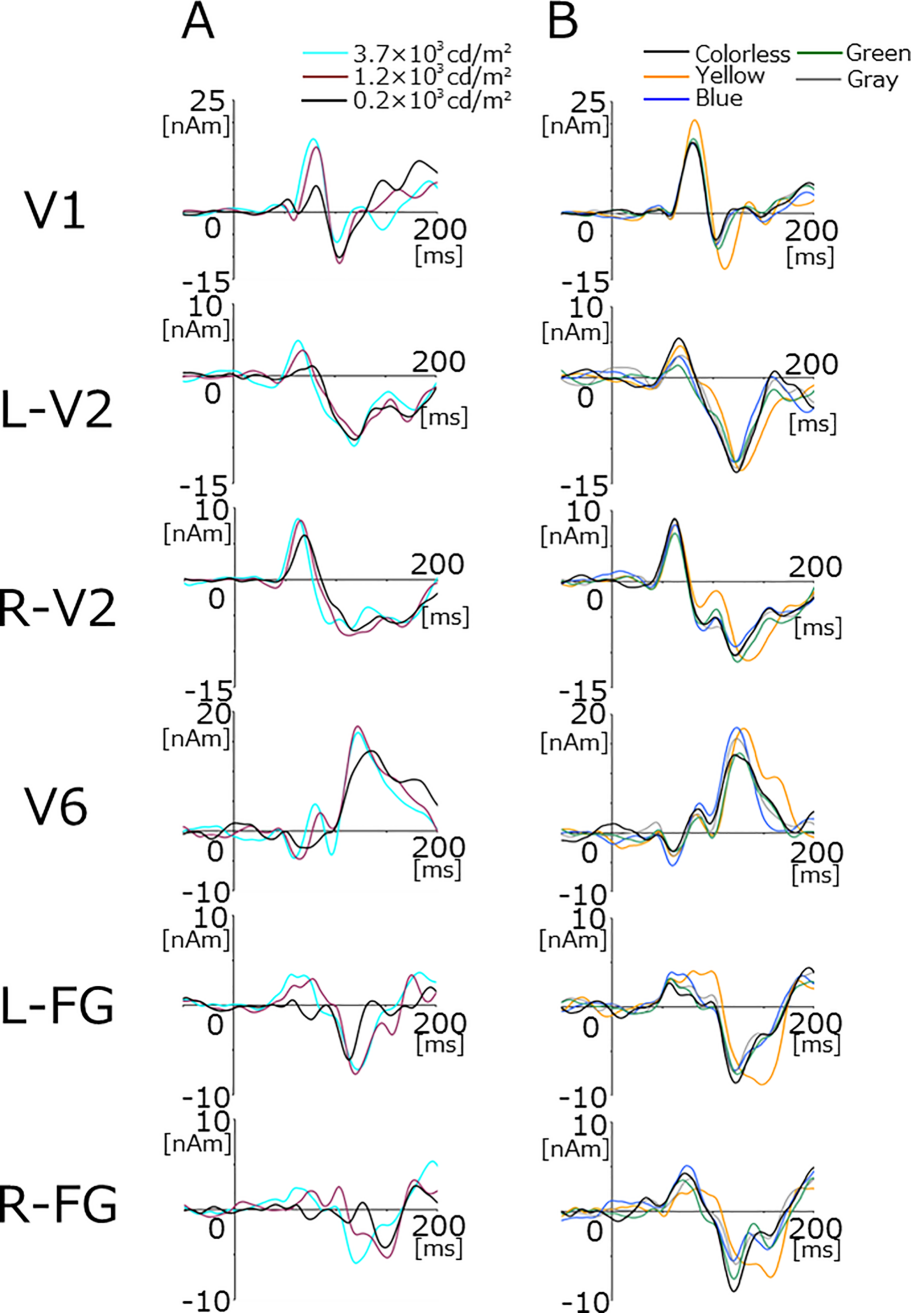
Grand-averaged source strength waveforms of each cortical activity. Waveforms for Experiment 1 (A) and Experiment 2 (B) are shown.

Although activated locations in the occipital area and the number of dipoles necessary to explain the evoked response slightly differed among subjects, dipoles were generally estimated to be located in the eye, primary visual cortex (V1), V2, V6, and fusiform gyrus (FG). V1 activity, with an upward current peaking at approximately 100 ms (M100), was estimated to be located in the calcarine fissure. The source responsible for the second major component at approximately 160 ms (M160) was located in the bilateral FG on the roof of the cerebellum. V2 sources pointing lateral in both hemispheres [12] were active before M100. A V6 source with an upward current and characteristic location in the midline of the parieto-occipital sulcus [9,12] was estimated in a few subjects. The mean Talairach coordinates of cortical sources are listed in Table 1. The grand-averaged waveforms of each source strength waveform are shown in Fig 4A. All of these source activities exhibited greater amplitudes and shorter response latencies for stronger light stimuli (Table 1).

**Table 1:** The mean latency and amplitude of each source activity in Experiment 1.

Source		Talairach coordinates				Latency (ms)			Amplitude (nAm)		
	N	x	y	z		3.7×10^3^	1.2×10^3^	0.2×10^3^	3.7×10^3^	1.2×10^3^	0.2×10^3^ (cd/m^2^)
L-retina	9	-18.9±5.0	73.3±8.6	-37.8±5.7	b-wave	67.2±5.3	80.1±7.5	91.0±13.5	11.4±3.7	6.9±2.3	0.91±3.4
R-retina	9	20.5±4.9	72.8±6.9	-40.6±9.4	b-wave	64.2±5.0	78.0±9.2	93.1±19.5	11.5±2.9	7.6±1.9	2.7±2.4
V1	10	2.3±7.2	-88.9±4.7	-7.4±8.6	M100	102.5±5.2	105.3±4.4	108.1±8.8	29.6±14.8	29.9±13.8	20.4±11.6
L-V2	9	-27.0±4.9	-80.7±9.0	-9.0±5.2	M110	106.1±15.7	107.0±16.7	108.7±14.4	20.6±11.6	16.3±8.8	14.0±7.6
R-V2	9	22.2±3.5	-83.1±7.1	-9.6 ± 4.0	M110	103.7±10.1	108.2±12.0	109.9±12.0	21.5±9.3	23.1±7.7	20.8±9.2
V6	5	-2.9±8.9	-78.6±6.8	23.0±10.4	M130	133.8±21.5	131.7±15.1	136.9±17.8	19.9±13.9	22.4±15.0	18.8±7.9
L-FG	7	-22.0±7.4	-77.0±12.8	-19.6±2.9	M160	159.9±10.2	161.6±13.2	164.6±11.7	2.5±10.8	5.0±8.4	3.1±5.2
R-FG	6	21.3±5.0	-78.6±11.4	-21.2±6.1	M160	154.5±3.0	155.7±8.1	153.1±9.0	1.9±8.6	7.2±9.6	6.0±8.0

FG, fusiform gyrus

### Experiment 2

**Fig 5:**
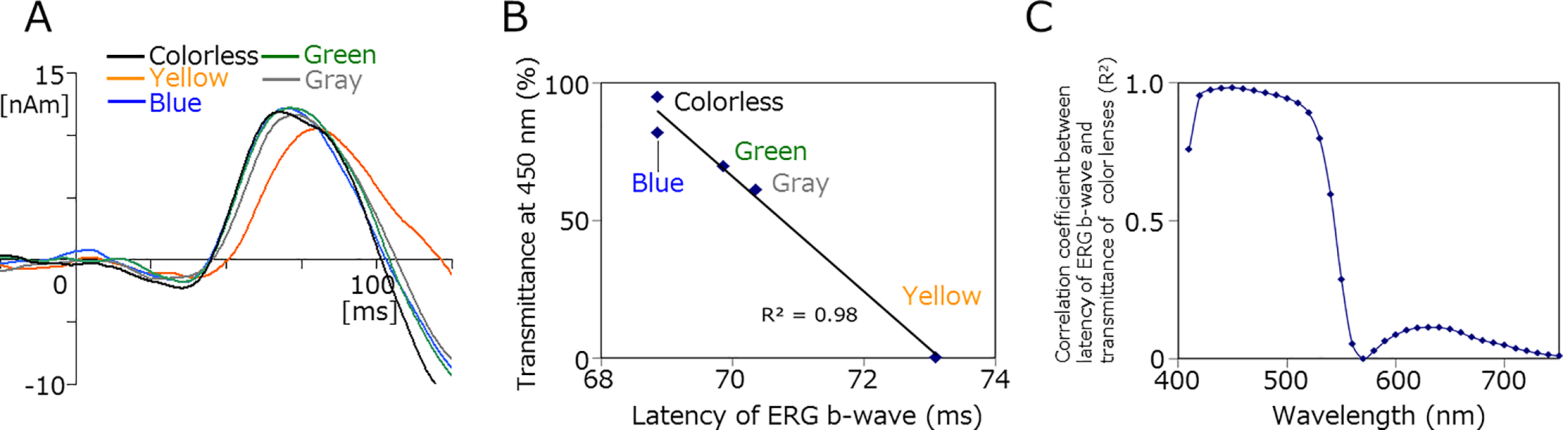
Correlation between b-wave latency and transmittance at 450 nm. A, Grand-averaged source strength waveforms of retinal activity in Experiment 2. The averaged waveforms of both eyes are shown. B, Relationship between the mean peak latency of retinal activity (b-wave) and transmittance of color lenses. C, Correlation coefficient plotted against wavelength.

**Fig 6:**
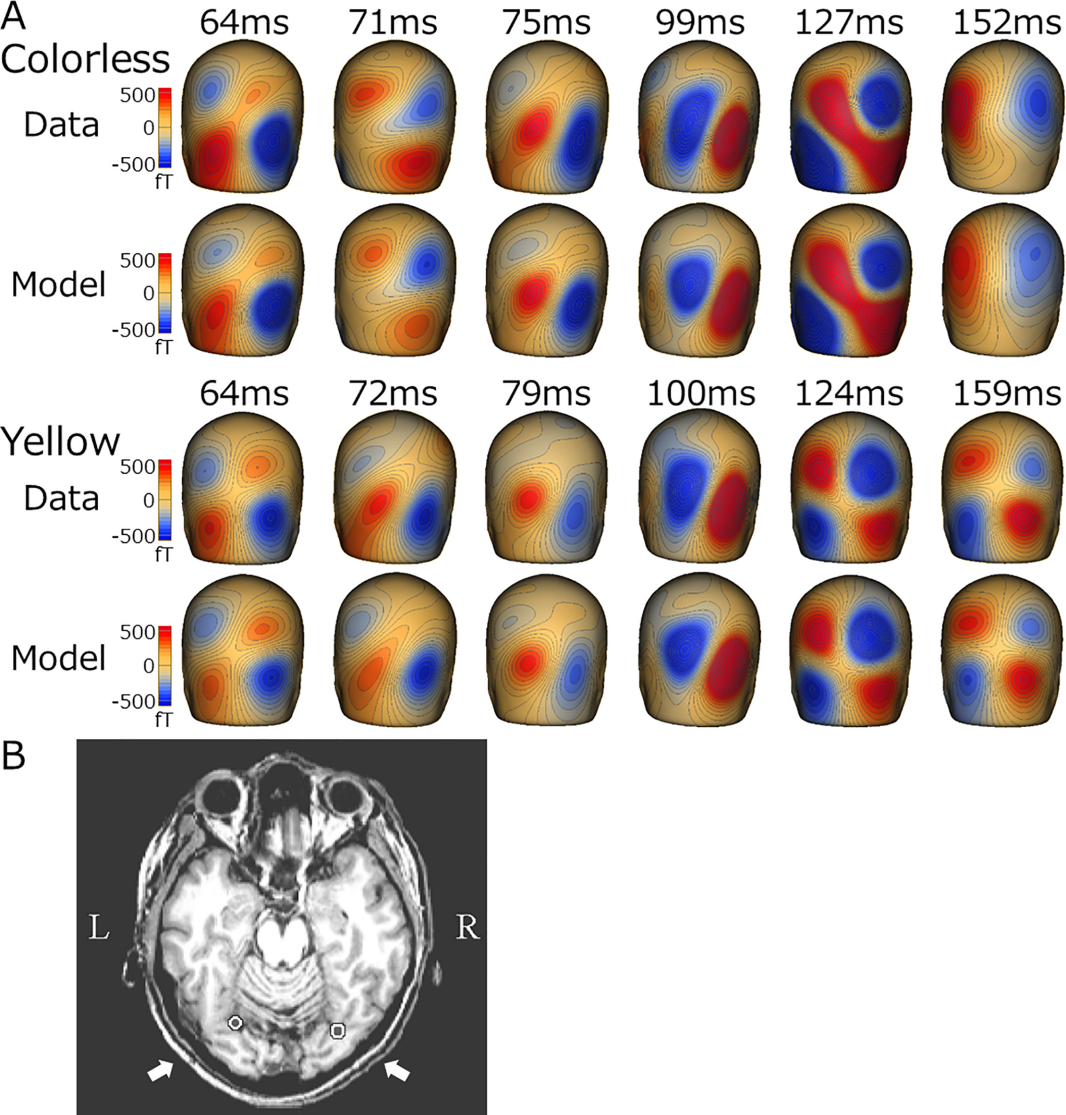
Effects of the yellow lens on VEF topography. Comparison of VEF topographies following a bright light stimulation between colorless and yellow lenses in a representative subject. Topographies for the recorded MEG signals (upper panel) and the model (lower panel) are shown. Note the similar topographies at early latencies, but lack of a clear quadrupole pattern distribution for the colorless lens condition at 150–160 ms. B, Dipole location for dipoles estimated in the fusiform gyrus region superimposed on a subject’s MR image.

Fig 4B shows grand-averaged source strength waveforms for each source activity. The yellow lens exerted specific effects on retinal and cortical responses (Figs 4B and 5). ANOVA results showed that the latency of the b-wave significantly differed among the five color lenses (F_(4,32)_ = 9.26, p = 4.3 × 10^−5^). Post hoc paired comparisons showed that the b-wave latency of the yellow lens was significantly longer than that of the colorless lens (*t*-test, p = 0.0022; Wilcoxon, p = 0.012) (Fig 5A, Table 2). The peak amplitude of the b-wave did not significantly differ among the lens conditions (F_(4,32)_ = 1.19, p = 0.34). However, when amplitude was measured at the peak latency for the colorless condition, it significantly differed among the five conditions (F_(4,32)_ = 5.82, p = 0.0013). Post hoc tests showed a significant difference between the yellow and colorless lenses (*t*-test, p = 0.034; Wilcoxon, p = 0.015), suggesting that viewing through the yellow lens reduced the amplitude of the early part of the b-wave, which prolonged peak latency. The latency of the b-wave strongly correlated with transmittance at 400–500 nm (Fig 5B and 5C), indicating that blue light contributed to shaping the early part of the b-wave. Despite the reductions induced in retinal activity by the yellow lens, some cortical activities were enhanced under this condition. ANOVA results showed that the effects of the lens color were significant on the amplitude of the source activities in V1 (F_(4.32)_ = 3.93, p = 0.01) and L-FG (F_(4,24)_ = 6.26, p = 0.0014). When compared with the colorless lens, post hoc tests revealed that only the yellow lens had significant effects on V1 (*t*-test, p = 0.024; Wilcoxon, p = 0.025) and L-FG (*t*-test, p = 0.018; Wilcoxon, p = 0.018) activities. R-FG activity was slightly enhanced by the yellow lens (8.4 nAm for the yellow lens vs 2.9 nAm for the colorless lens, *t*-test, p = 0.13; Wilcoxon, p = 0.12). Regarding peak latency, the effects of the lens color was significant for V1 (F_(4,32)_ = 8.08, p = 0.00013). Similar to the effects on amplitude, only the V1 latency of the yellow lens differed significantly from that of the colorless lens (*t*-test, p = 0.024; Wilcoxon, p = 0.012). Fig 6 shows a comparison of field distributions between colorless and yellow lens conditions in a representative subject. In this case, the field distribution was very similar between the two conditions with the exception that a characteristic quadrupole pattern distribution was present at 150–160 ms for the yellow lens condition only. Neural sources were estimated to be located in FG in both hemispheres (Fig 6B).

**Table 2:** The mean latency and amplitude of each source activity in Experiment 2.

Source			Latency (ms)					Amplitude (nAm)				
	N		Yellow	Blue	Gray	Green	Colorless	Yellow	Blue	Gray	Green	Colorless
L-retina	9	b-wave	83.0±12.6*	70.5±7.3	72.9±6.6	71.9±4.3	69.9±6.5	12.0±3.3	13.0±5.6	13.3±5.9	12.6±4.5	13.5±4.5
R-retina	9	b-wave	83.3±12.2*	71.8±5.1	73.7±9.2	69.7±5.1	70.1±6.5	11.3±2.8	12.5±2.8	13.1±5.3	12.8±5.0	12.9±3.5
V1	10	M100	111.6±5.0*	106.2±5.7	105.7±5.0	106.1±5.4	105.5±6.3	37.1±14.5*	32.6±20.1	31.5±22.6	33.5±21.0	30.2±19.3
L-V2	9	M110	126.9±19.2	122.3±18.1	124.2±17.4	123.5±19.3	123.7±19.4	23.4±14.0	11.1±16.4	19.0±12.1	18.2±11.0	21.5±14.8
R-V2	9	M110	116.5±19.6	115.2±17.1	114.4±18.5	114.4±16.7	113.5±19.6	26.0±13.1	24.4±11.0	25.1±11.5	25.0±12.1	26.3±12.5
V6	5	M130	129.1±4.7	123.3±8.0	123.1±7.1	123.1±7.1	121.7±9.1	18.3±11.4	21.3±20.2	18.7±17.0	15.7±8.8	17.5±11.4
L-FG	7	M160	156.8±7.1	157.2±10.2	158.5±8.3	157.4±9.4	161.4±9.4	10.5±12.3*	4.8±10.6	4.1±10.5	5.3±8.4	5.0±10.0
R-FG	6	M160	157.5±8.1	154.0±7.2	162.5±13.4	155.5±7.5	161.7±5.7	8.4±4.4	4.9±5.4	4.5±6.5	4.8±3.7	2.9±8.3

*, p < 0.05 vs colorless.

## Discussion

### Retinal and cortical responses

Since the yellow lens had the largest impact, at least some of the present retinal activity observed originated from cones sensitive to short wavelengths (S-cones). On the other hand, the result showing that the blue or green lens had negligible effects on retinal activity suggested the lesser contribution of middle (M) and long (L) wavelength-sensitive cones. It currently remains unclear which cells contributed to shaping the response observed under the yellow lens condition with a longer response latency (Fig 6A). The response with the colorless lens was considered to be mainly composed of rod and S-cone components. Under the yellow lens condition, the S-cone component was reduced, which resulted in a longer implicit time for the b-wave. M- or L-cone ERGs have shorter latencies than S-cone ERGs, while S-cone ERGs have shorter latencies than rod ERGs [13,14]. Although the bright light was repeatedly presented at least 100 times under each condition in the present study, light adaptation may not have been complete because we used a black background. In a previous study using multifocal ERGs, Hoffmann et al. [15] reported minor effects of blue-light filtering. Although the methodologies used in their study and the present study differed, the range of filtering appeared to be the most important factor. The transmittance of the 450-nm wavelength in the present study was almost 0%, but was approximately 50% in their study. Therefore, a wavelength range of 400–530 nm, in which the present transmittance was clearly lower than theirs, appeared to be responsible for the reduced and delayed retinal response.

The beneficial effects of blue-light filtering on visual function have been reported in previous studies on contrast sensitivity [16–19], brightness [20], glare [21–25], reaction time [26], and scattering (described below) and VEPs in monkeys [27]. Previous findings also support the wavelength of light affecting the emotional aspects of photophobia. Main et al. [28] examined the discomfort threshold to light of short, medium, and long wavelengths in patients with migraine, patients with tension-type headache, and healthy controls, and found that patients with migraine had a significantly lower threshold for short and long wavelengths than the other two groups. Other studies reported that patients with photophobia preferred yellow tinted lens [29]. In the present study, cortical activities in V1 and FG were increased by the yellow lens in spite of reduced retinal activity. Since these cortical areas are in the ventral pathway of visual processing, this result suggested a functional change related to object recognition when bright light was viewed through a yellow lens, and, thus, the combination of reduced retinal activity and enhanced activity in the visual ventral pathway may be an objective marker for these functional changes. A case study by Horiguchi et al. [30] describing three patients with the total lack of photophobia due to bilateral lesions in the ventral occipital lobe appears to support this notion.

### Effects of wavelength

This combination was specifically observed when subjects wore the yellow lens, implying that short wavelength light contributed to the disturbance of vision. Several mechanisms have been suggested to contribute to the results obtained in the present study. Bright light causes uncomfortable sensations (discomfort glare) as well as vision impairments (disability glare) [31]. Disability glare is caused by scattered intraocular light that casts a veiling luminance on the retina and, thus, reduces image contrast and chromatic discrimination [32]. Scattered light in the cornea and lens is predominantly of a short wavelength [33] and is partly due to small particles [34]. Therefore, the effects of the yellow lens on retinal and cortical activities in the present study may come from reduced light scattering. In the presence of glare, previous clinical studies showed that a yellow intra-ocular lens (IOL) improved driving performance [21,22] as well as recovery from photostress by a bright flash [23,25]. In 150 young healthy subjects, Hammond et al. [24] examined glare disability, photostress recovery, and their relationship with macular pigmentation using intense white light. They found that macular pigmentation correlated with these visual variables, which supported yellow intraocular filters enhancing chromatic contrast, reducing glare discomfort and dazzle, and thereby enhancing detail by absorbing blue haze [35]. Lenticular fluorescence also causes light scattering and affects visual function [27,36–39]. Lenticular fluorescence occurs due to the emission characteristics of certain compounds associated with lens proteins and has been shown to originate from chromophores with activation wavelengths of approximately 340–360 nm (emission at 420–440 nm) and 420–435 nm (emission at 500–520 nm) [27,38–42]. In the present study, the transmittance of light of this wavelength range markedly differed between the yellow and other lenses.

The second possible mechanism is chromatic aberration. Since lenses do not focus different wavelengths of light in a point, the retinal image formed by white light is blurred. Therefore, light scattering described above and chromatic aberration may cause a blur in the retina, and, thus, blue-light filtering is expected to improve contrast sensitivity. Campbell and Gubisch [43] measured contrast thresholds for monochromatic yellow light and white light, and observed a lower threshold for the former for intermediate frequencies. Similar effects on the contrast sensitivity of blue-light filtering were demonstrated in subjects with intraocular lenses [17,19]. Wolfesohn et al. [18] reported that yellow lenses reduced contrast sensitivity to a white-on-black grating and enhanced it to a white-on-blue grating in low to mid-range spatial frequencies. However, a study by Kelly et al. [44] using 52 healthy young subjects found no significant differences in contrast sensitivity between yellow and neutral lenses. Blue-light filtering generally appears to exert positive effects on contrast sensitivity under photopic [16,17] or mesopic conditions [16] at middle-range spatial frequencies [17], but not at high frequencies [45].

The third possibility is the color opponent system. Kinney et al. [26] measured reaction times for the square wave gratings of various spatial frequencies and contrasts viewed through yellow and neutral goggles, and observed that the yellow goggle increased reaction times to low-luminance gratings of middle frequencies. They suggested that the beneficial effects of the yellow lens came from enhancements in the output of the chromatic system due to reductions in subtractive effects in the yellow-blue opponent channel. Their findings showing that the yellow lens did not affect reaction times to gratings of high frequencies were consistent with those in previous studies showing no effects of a yellow lens on visual acuity [45], and precluded simple explanations for the effects of yellow lenses, such as a reduction in scattering light [26]. These non-optical factors may have contributed to the effects of the yellow lens observed in the present study.

Although the present study did not clarify the extent to which these mechanisms contributed to the results obtained, the findings of a VEP study in rhesus monkeys by Zuclich et al. [27] are noteworthy. They showed that the measured fluorescence of the lens was sufficiently high to imply the degradation of visual function, and in order to confirm this, they presented a 413-nm laser beam to the monkey’s eye while recording VEPs to monochromatic sine-wave gratings. The findings obtained showed that the amplitude of VEPs decreased by 15~25% when 413-nm wavelength light was projected, which they attributed to the veiling glare associated with the fluorescence induced because under the experimental conditions, a direct glare with light scattering of 413 nm did not explain the decline. Van den Berg stated that a value of approximately 20% for the VEP amplitude reduction matched the expected value of veiling luminance caused by 413-nm wavelength light calculated from excised human lenses [38]. To the best of our knowledge, there is one VEP study that investigated the effects of blue-light filtering in humans [46]. In contrast to the present results, they found no effects on VEP amplitudes. This difference may be attributed to the yellow lens used; we used a yellow lens that cut off light with a wavelength shorter than 450 nm (Fig 2), while transmittance at 450 nm of the IOL used by Hoffmann et al. was approximately 50%. Another study also failed to show a significant effect of the same yellow lens on ERGs [15]. Therefore, light with wavelengths of 380–530 nm, at which transmittance in the two studies clearly differed, appeared to have an impact on retinal and cortical activities. This was in agreement with the findings of Zuclich et al. [27] showing that a wavelength of 413 nm was effective.

## Conclusions

The present results demonstrated that retinal activity (b-wave) in response to bright light was reduced when subjects viewed it through a yellow lens, while cortical activities in V1 and FG were enhanced, suggesting changes in visual processing by blue-light filtering. It is interesting to note that Good and Hou reported that children with cortical or cerebral visual impairment (CVI), which often accompanies photophobia, exhibited better visual acuity under low- than high-luminance viewing conditions, while no significant differences were observed between the two conditions in normal children [47]. In addition, the amplitude of VEP to low-luminance grating was greater than that to high-luminance in the CVI group, which is consistent with the present results. Therefore, blue-light filtering may be beneficial for these patients. The present results may be useful for selecting the color and depth of color lenses; however, future clinical studies are warranted.
